# Green Preparation, Structural Characterization, and Antioxidant Activity of Apple Pectin Oligosaccharides Obtained by Hydrogen Peroxide Degradation

**DOI:** 10.3390/molecules31111923

**Published:** 2026-06-03

**Authors:** Huipeng Liu, Junhao Li, Shanyu Xie, Xiang Su, Xihuang Lin, Yunli Cao

**Affiliations:** 1 College of Chemistry and Chemical Engineering, Pingdingshan University, Pingdingshan 467000, China; 231017092@e.pdsu.edu.cn (J.L.); suxiang160029@163.com (X.S.); 2 National Biochemical Engineering Technology Research Center, Nanjing Technology University, Nanjing 211816, China; 3 Department of Chemical and Biochemical Engineering, College of Chemistry and Chemical Engineering, Xiamen University, Xiamen 361005, China; 20620220156500@stu.xmu.edu.cn; 4 Analysis and Test Center, Third Institute of Oceanography, Ministry of Natural Resources, Xiamen 361005, China

**Keywords:** heteropolysaccharide, alkaline oxidative hydrolysis, structural elucidation, oligoglycans, radical scavenging ability

## Abstract

Pectin oligosaccharides are promising natural bioactive ingredients, while traditional preparation methods suffer from harsh conditions and environmental drawbacks. Herein, an eco-friendly alkaline hydrogen peroxide degradation method was applied to produce apple pectin oligosaccharides (APOSs), followed by ethanol precipitation. The structural features and antioxidant potential of APOSs were comprehensively characterized. Galacturonic acid (58.2%) was determined as the dominant monosaccharide, along with minor galactose, arabinose, and rhamnose. Sixteen oligosaccharides consisting of homogalacturonan fragments and RG-I hetero-oligosaccharides were identified using NMR, FTIR and UPLC/Q-TOF-MS. In vitro assays demonstrated excellent radical-scavenging capacities of APOSs. The DPPH and ABTS scavenging rates reached 84.6% (10 mg/mL) and 96.2% (9 mg/mL), respectively. This study provides a feasible green strategy for APOS fabrication and clarifies its antioxidant potential, supporting the industrial application of APOS as a high-efficiency natural antioxidant in functional foods.

## 1. Introduction

Pectin is a ubiquitous and structurally intricate macromolecule in plant cell walls. It is mainly composed of galacturonan-rich domains with various neutral sugar side chains, which confer pectin with versatile physicochemical properties and biological activities [1]. However, natural pectin is characterized by high molecular weight, high viscosity, and low water solubility [2]. Through depolymerization, pectin can be degraded into lower molecular weight carbohydrate products, known as pectic oligosaccharides (POSs) [3]. According to different criteria, POSs are formed by 2–50 monomeric units or can show molecular weight (Mw) values up to 5–7 kDa [4,5]. Compared to pectin, POSs exhibit significant advantages in terms of biological activity expression due to their lower molecular weight, better water solubility, and higher bioavailability, thereby holding broader application prospects in the food, pharmaceutical, and biomaterial fields [6].

In recent years, the antioxidant activity of POSs has garnered extensive attention. In the food industry, POSs can be incorporated as natural antioxidants into lipid-containing products, dairy products, or meat products, effectively delaying lipid oxidation and pigment degradation, thereby extending product shelf life [7,8,9]. In the pharmaceutical field, POSs can alleviate oxidative stress and protect the structural and functional integrity of cell membranes through mechanisms such as scavenging free radicals, chelating metal ions, or activating endogenous antioxidant systems [10]. These properties highlight their promising potential as functional foods or adjunct therapeutic agents. The antioxidant activity of POSs is closely associated with their molecular structure. Studies have shown that factors such as the charge density and distribution of the sugar chain (e.g., the proportion of free carboxyl groups from galacturonic acid), the type and linkage pattern of neutral sugar side chains (e.g., the degree of branching of galactose and arabinose), and the cleavage pattern of glycosidic bonds significantly influence the free radical-scavenging capacity of POSs and their interaction mechanisms with target molecules [11,12]. This structure–activity relationship provides a theoretical basis for the structural modification and functional enhancement of POSs.

Various techniques are available for producing POSs with distinct structural features, which can be categorized into physical [13], chemical [14], and enzymatic approaches [15]. Physical methods tend to generate polysaccharide fragments with uneven molecular weight distribution and poor reproducibility, making them unsuitable for producing defined oligosaccharides. Moreover, these techniques require highly specialized equipment, which restricts their application to laboratory-scale research and prevents industrial implementation [16]. For chemical strategies, acid hydrolysis remains a common practice, utilizing strong acids such as trifluoroacetic acid [17], hydrochloric acid [18], and nitric acid [19]. However, this method has obvious limitations. Neutral sugars such as galactose and arabinose are easily degraded under mild acid conditions, while cleavage of the homogalacturonan (HG) domain requires high temperatures and harsh environments [20]. Furthermore, concentrated acids produce large amounts of wastewater and cause potential environmental risks [21,22]. By contrast, enzymatic hydrolysis is characterized by high substrate specificity, excellent selectivity, mild operational conditions, and environmental compatibility [23]. Nevertheless, this method has a higher cost compared to the chemical method [21]. In contrast, the hydrogen peroxide degradation method, as a green chemical process, features mild reaction conditions (typically carried out at moderate temperatures and near-neutral pH), with water and oxygen as the final decomposition products and no toxic residues, thereby demonstrating significant advantages in the green preparation of POS [24].

Apples are one of the most extensively cultivated fruits, with abundant nutritional ingredients and diverse health-beneficial properties. Globally, approximately 4 million tons of apple pomace are produced each year as major by-products during fruit deep processing, which are inexpensive and easily accessible in large quantities [25]. At the present time, commercial production of pectin is limited to two major sources, apple pomace and citrus peel. Nowadays, the largest part of commercially available pectin originates from citrus peel (85.5%), and only a small proportion is covered by extraction from apple pomace (14.0%) and sugar beet pulp (0.5%) [26]. Pectin isolated from apple pomace has been widely exploited in food, pharmaceutical, and cosmetic fields [26]. Our previous studies have mainly focused on citrus pectin [27], while fewer systematic investigations have been conducted on apple pectin and its derived oligosaccharides. Therefore, apple pectin was selected as the research substrate in this work. Apple pectin oligosaccharides (APOSs) were successfully prepared through hydrogen peroxide-assisted degradation coupled with ethanol fractional precipitation. Furthermore, the structural features of obtained APOS were comprehensively characterized, and its in vitro antioxidant capacity was determined via DPPH and ABTS radical scavenging methods. This study aims to lay a theoretical foundation for the green and high-efficiency preparation of APOS, and promote its further development and utilization in antioxidant functional food and related products.

## 2. Results and Discussion

### 2.1. Monosaccharide Composition Analysis of APOS

**Figure 1:**
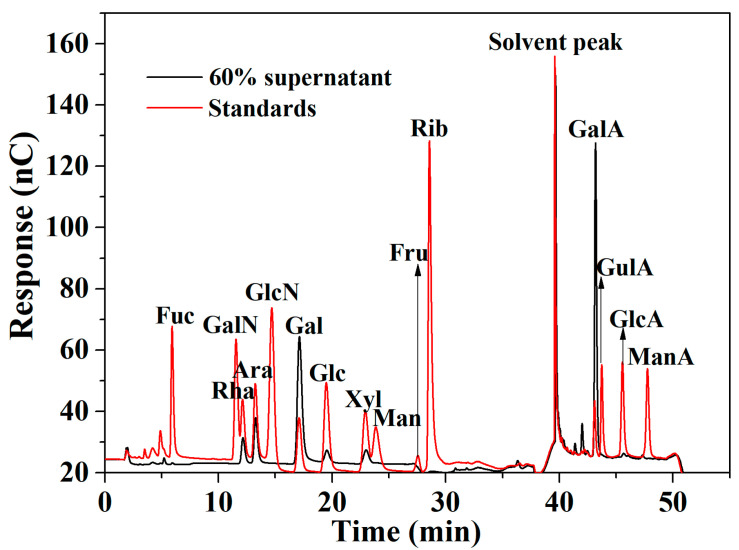
Chromatographic profile of monomeric composition of APOS. (Fuc: Fucose, GalN: Galactosamine, GlcN: Glucosamine, Man: Mannose, Fru: Fructose, Rib: Ribose, GulA: Guluronic acid, ManA: Mannuronic acid, Rha: Rhamnose, Ara: Arabinose, Gal:Galactose, Glc: Glucose, Xyl:Xylose, GalA: Galacturonic acid, GlcA: Glucuronic acid).

As shown in Figure 1 and Table 1, the monosaccharide composition of APOS was dominated by galacturonic acid (GalA, 58.2%), galactose (Gal, 28.6%), arabinose (Ara, 4.5%), and rhamnose (Rha, 4.1%), along with low amounts of xylose (Xyl, 2.6%), glucose (Glc, 1.6%), and glucuronic acid (GlcA, 0.3%). Glc and GlcA were mainly derived from non-pectic polysaccharides [27,28]. Compared with original apple pectin [29], APOS prepared by alkaline hydrogen peroxide depolymerization combined with ethanol separation showed a decreased content of GalA and an increased content of Gal. Consistent with our results, Hu et al. also found that pectin depolymerized via the synergistic effect of sodium bicarbonate, hydrogen peroxide and ultrasound presented a reduction in GalA content and an elevation in Gal level [30]. Traditional enzymatic hydrolysis of pectin for preparing pectin oligosaccharides requires dialysis separation to obtain fragments rich in galacturonic acid and galactose, accompanied by a high content of neutral sugars [31]. In this study, fragments with similarly high proportions of galacturonic acid and galactose were successfully prepared via alkaline oxidative degradation. This strategy effectively avoids the high cost of enzymes, the difficult post-treatment of homogeneous enzymatic catalysts, and complicated purification procedures. The molar ratio of Rha/GalA was 0.07, indicating that APOS was mainly derived from HG of apple pectin. The previous results indicated that hydroxyl radicals showed high reactivity toward HG regions, yielding oligogalacturonides [32]. The molar ratio of (Gal+Ara)/Rha of APOS was 8.07, indicating that APOS contained the oligosaccharides from the RG of apple pectin. The oligosaccharides from the RG were detected via NMR and UPLC-/Q-TOF-MS.

### 2.2. SEM Analysis of Apple Pectin and APOS

**Figure 2:**
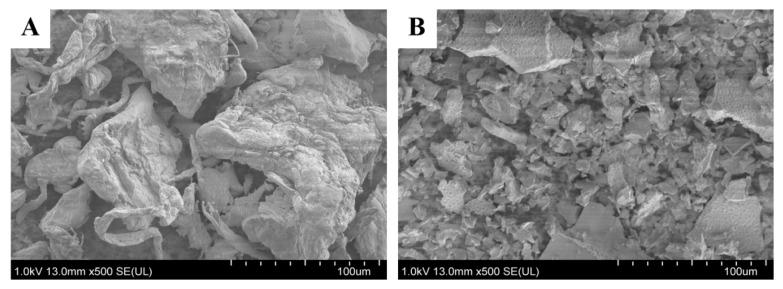
SEM images of (**A**) apple pectin and (**B**) APOS.

Figure 2 displays scanning electron micrographs of apple pectin and APOS obtained at 500× magnification. As observed in the SEM images, native apple pectin exhibits an irregular block-like morphology with a relatively rough surface, which is consistent with previous observations reported by Choudhury et al. [33]. In contrast, APOS appears as fine particles and fragmented debris. The formation of these small particles reflects a disrupted and fragmented structure, implying that hydrogen peroxide treatment promotes the cleavage of glycosidic linkages, thereby inducing the degradation of pectin macromolecules and the generation of low-molecular-weight pectin oligosaccharides [33]. These results are in good agreement with the findings reported by Su et al. [27].

### 2.3. FTIR Analysis of Apple Pectin and APOS

**Figure 3:**
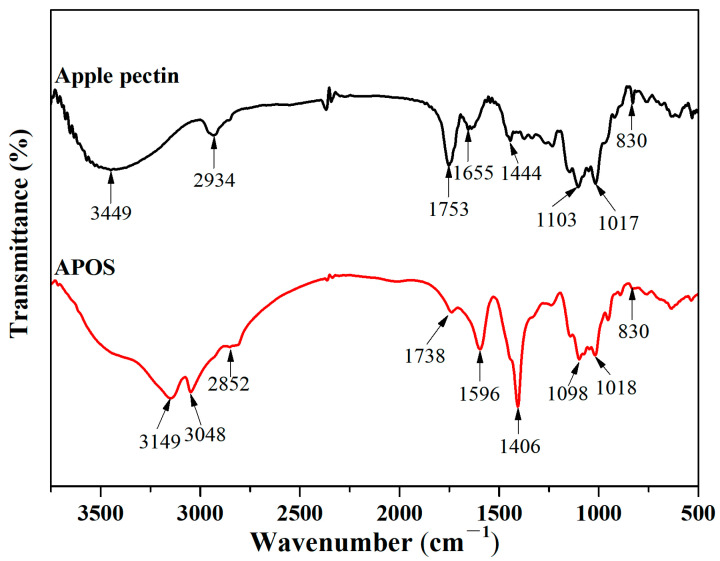
FTIR spectra of apple pectin and APOS.

The FTIR spectra of apple pectin and APOS (Figure 3) both exhibit characteristic absorption peaks. The broad and strong absorption peak at ~3449 cm^−1^ in apple pectin, corresponding to the stretching vibration of O–H bonds from sugar residues and hydrogen bonds, shifts to lower wavenumbers and splits into two peaks at 3149 cm^−1^ and 3048 cm^−1^ in APOS [34]. The possible reason might be the rearrangement of the intra- and inter-molecular hydrogen-bonding microenvironment. The de-esterification increased the content of free carboxyl groups, making hydroxyl groups in different hydrogen-bonding states exhibit distinct vibration frequencies, thus leading to peak splitting. Similar spectral phenomenon and mechanisms of hydrogen bond rearrangement for galactaric acid have been reported in previous FTIR study [35]. The C–H stretching vibration peak at 2934 cm^−1^ in apple pectin weakens significantly in APOS, while a new weak peak at 2852 cm^−1^ appears, which is attributed to the symmetric C–H stretching vibration of –CH_2_ groups [36]. For carbonyl and carboxyl groups, the ester bond C=O stretching vibration peak at 1753 cm^−1^ in apple pectin shifts to 1738 cm^−1^ with reduced intensity in APOS, while the carboxylate (–COO^−^) asymmetric stretching peak at 1655 cm^−1^ red-shifts to 1596 cm^−1^ and becomes stronger, accompanied by the carboxylate symmetric stretching/C–H bending peak at 1444 cm^−1^ shifting to 1406 cm^−1^ with enhanced intensity, collectively demonstrating that APOS undergoes de-esterification with increased free carboxyl groups during degradation [34,37]. In alkaline systems, pectin is prone to saponification and demethylation reactions, which serve as the core cause of this phenomenon. In the carbohydrate fingerprint region, both samples show characteristic pyranose ring and glycosidic linkage peaks at ~1100–1017 cm^−1^ (1103/1017 cm^−1^ for apple pectin, 1098/1018 cm^−1^ for APOS), indicating retention of the pyranose skeleton [37,38]. Additionally, the absorption peak at 830 cm^−1^ in both spectra suggests the presence of the α-configuration in the sugar residues, with no change in anomeric configuration during degradation [39].

### 2.4. NMR and UPLC/Q-TOF-MS Analysis of APOS

**Figure 4:**
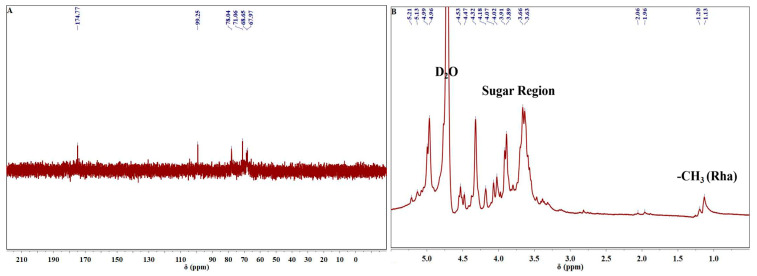
(**A**) ^13^C NMR spectra and (**B**) ^1^H NMR spectra of APOS.

Nuclear magnetic resonance (NMR) spectroscopy can be applied to identify the types of sugar residues and glycosidic substitution positions of polysaccharides [40]. Figure 4 presents the ^1^H and ^13^C NMR spectrum of APOS, and characteristic chemical shifts were systematically assigned to clarify its structural features. The signals at δ174.77 ppm and δ99.15 ppm are assigned to the C6 carboxyl carbon and anomeric C1 of methyl esterified GalA, respectively [41]. Meanwhile, the characteristic peaks at δ69.6 ppm, δ69.9 ppm, δ78.4 ppm and δ72.6 ppm correspond to the C-2, C-3, C-4 and C-5 ring skeleton carbons of the → 4)-GalpA-(1 → sugar residue [42]. The proton signals of most sugar rings in APOS concentrated in the range of δ3.63–5.22 ppm. The anomeric proton signals distributed at δ4.60–5.13 ppm confirmed the coexistence of α- and β-glycosidic bond configurations [43]. The signals at δ5.02 ppm (non-esterified α-(1 → 4)-D-GalA) and δ4.96 ppm (methyl-esterified α-(1 → 4)-D-GalA-6-OMe) confirmed structural heterogeneity of the homogalacturonan (HG) backbone [44]. Characteristic resonances at δ3.72, δ4.09, δ4.48, and δ4.64 ppm were H-2, H-3, H-4, and H-5 of 1,4-linked α-GalA, respectively [45]. The signal around δ3.63 ppm was assigned to methyl protons of methyl-esterified GalA residues [46]. Additionally, typical methyl signals of α-L-rhamnopyranosyl residues ((→2)-α-L-Rhap-(1→)) were detected at δ1.11/1.19 ppm, while acetyl group signals appeared at δ1.94/2.05 ppm [40]. The resonance at δ5.22 ppm was preliminarily attributed to the anomeric H-1 of → 3,5)-α-Araf-(1 → [41]. Weak signals around δ2.00 ppm were found, suggesting that APOS contained a very small amount of O-acetyl groups [47,48]. Comprehensive analysis of the NMR spectrum further demonstrated that APOS was mainly composed of oligosaccharide fragments derived from the HG domain of pectin, which was highly consistent with the above monosaccharide composition results.

**Figure 5:**
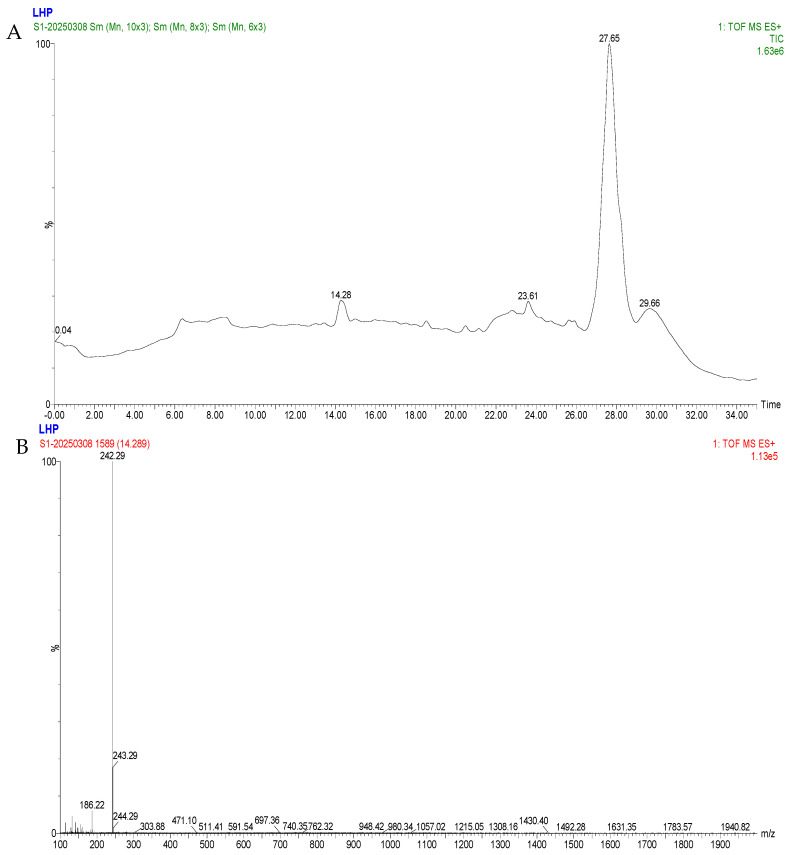

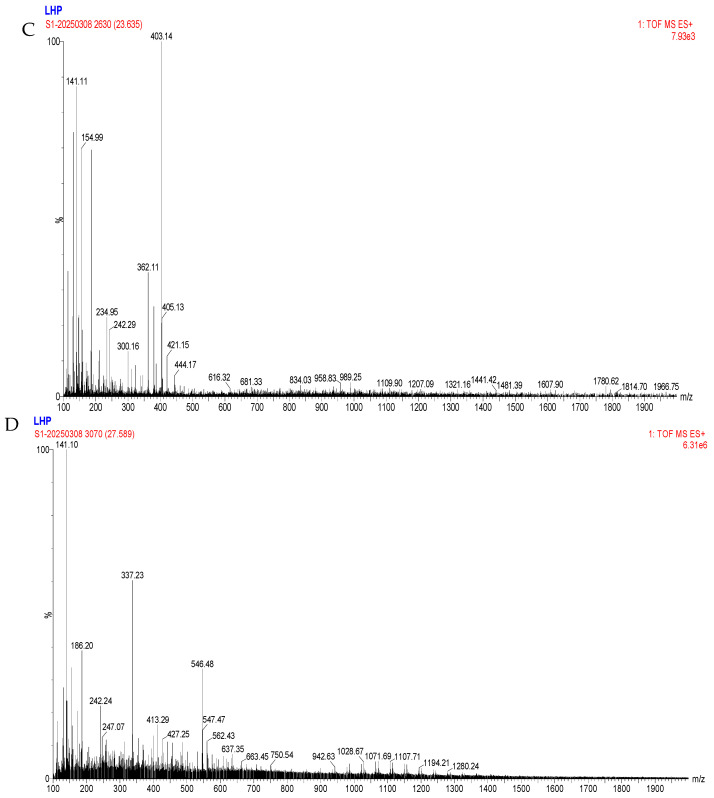
Ion chromatogram (**A**) and MS spectra ((**B**): 14.28 min, (**C**): 23.61 min, (**D**): 27.45 and 29.66 min).

Complementary to NMR qualitative structural identification, mass spectrometry (MS) further supplemented molecular weight distribution, actual degree of polymerization, and specific oligosaccharide fragment information, realizing mutual verification and integrated structural characterization [27]. The ion chromatogram and MS spectra of APOS are displayed in Figure 5. Combined with Glycoworkbench analysis [49], the maximum degree of polymerization of identified APOS fragments reached DP 8 (Table 2), and methyl-esterified as well as acetylated galacturonic acid structural units were successfully identified, which was fully consistent with the substitution characteristics concluded from NMR spectral assignment. This structural feature was also in accordance with the previous research results reported by Remoroza et al. [50].

The identified oligosaccharide fragments in Table 2 were derived from both homogalacturonan (HG) and rhamnogalacturonan I (RG-I) domains, and exhibited distinct structural stability under alkaline H_2_O_2_ conditions. The reaction system in this study maintained an alkaline environment, which promoted the de-esterification of high-methoxyl apple pectin and reduced the steric hindrance of the linear HG backbone [51]. As reported in previous research, reactive oxygen species generated in alkaline oxidation systems preferentially attack the galacturonic acid backbone of the HG domain [30]. In contrast, acidic media tends to destroy the neutral sugar side chains of RG-I, while an alkaline environment causes little damage to these branched structures [51]. As a result, the HG domain was preferentially and extensively degraded, while RG-I fragments were well preserved under the alkaline H_2_O_2_ treatment. This degradation pattern is consistent with previously reported structural behavior of pectin in alkaline hydrogen peroxide systems [30].

### 2.5. Antioxidant Activity of APOS

**Figure 6:**
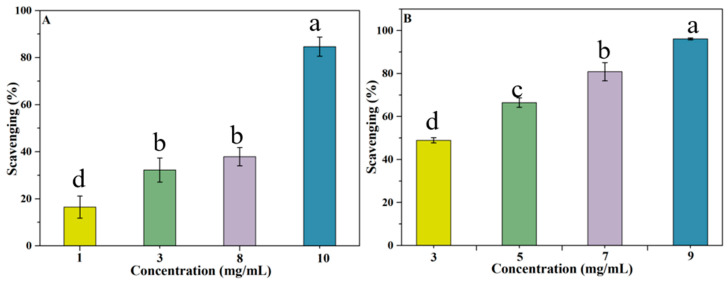
Concentration-dependent (**A**) DPPH and (**B**) ABTS radical scavenging rate (%) of the APOS. Data represent mean ± standard deviation (SD) of triplicate determinations. Values followed by different lowercase letters (a–d) are significantly different at *p* < 0.05 according to Duncan’s range test.

The results of DPPH and ABTS scavenging activity of APOS are shown in Figure 6. The antioxidant activity of APOS increased with APOS concentration. The calculated IC_50_ values were 6.86 mg/mL for DPPH assay and 3.09 mg/mL for ABTS assay. The DPPH and ABTS scavenging activity of APOS was 84.6% (10 mg/mL) and 96.2% (9 mg/mL). Enzyme hydrolyzed oligosaccharides from potato peel showed 77.52% (10 mg/mL) DPPH scavenging activity and 100% (10 mg/mL) ABTS scavenging activity [43]. The higher DPPH scavenging activity of APOS may be attributed to the presence of a greater amount of oligogalacturonic acid and a lower molecular weight. Related studies have shown that low-molecular-weight pectin oligosaccharides possess stronger antioxidant activity than the original pectin [22]. Previous research has verified that high-purity oligogalacturonic acid exhibits excellent and superior antioxidant activity even at low concentrations [44]. In terms of the underlying antioxidant mechanism, polysaccharide antioxidant capacity is closely correlated with the hydroxyl groups located at the C-2, C-3, and C-6 positions of the pyranose ring. High-molecular-weight polysaccharides usually exhibit compact molecular conformations and intensive intramolecular hydrogen bonds, which restrict the exposure of active hydroxyl groups and consequently weaken radical-scavenging efficiency. By contrast, low-molecular-weight oligosaccharides possess loose molecular structures, more exposed free hydroxyl groups, larger specific surface areas, and better water solubility, thereby facilitating efficient interactions with free radicals [52]. Therefore, in future research, more refined separation and purification technologies should be adopted to further separate and purify APOS components with different molecular weights and degrees of polymerization.

## 3. Materials and Methods

### 3.1. Materials

Apple pectin (Product No. 93854) was obtained from Sigma-Aldrich (Shanghai) Trading Co., Ltd., Shanghai, China. Ammonia water of analytical grade, anhydrous ethanol, and 30% hydrogen peroxide were purchased from Zhengzhou Paini Chemical Reagent Factory, Zhengzhou, China. 1,1-Diphenyl-2-picrylhydrazyl (DPPH) and 2,2′-azinobis(3-ethylbenzothiazoline-6-sulfonic acid) diammonium salt (ABTS), along with heavy water (D_2_O), were supplied by Shanghai Aladdin Biochemical Technology Co., Ltd., Shanghai, China. Trifluoroacetic acid (AR, Batch No. A0356762), sodium acetate (GR, Batch No. 191126), and 50% sodium hydroxide solution (GR, Batch No. Z21E036) were acquired from ACROS Organics, Geel, Belgium, Thermo Fisher Scientific, Waltham, MA, USA, and Alfa Aesar, Ward Hill, MA, USA, respectively. A series of monosaccharides and uronic acids, including mannose, rhamnose, galacturonic acid, galactose, glucose, glucuronic acid, arabinose, xylose, fucose, glucosamine hydrochloride, N-acetyl-D-glucosamine, D-fructose, D-ribose, galactosamine hydrochloride, L-guluronic acid (≥98%), and D-mannuronic acid (≥98%), were all of analytical grade and purchased from Boruaitang Biological Technology Co., Ltd., Yangzhou, China. Potassium bromide (AR, P116270) was supplied by Shanghai Aladdin Biochemical Technology Co., Ltd., Shanghai, China. Chromatographic grade acetonitrile and formic acid were obtained from Merck (China) Co., Ltd., Shanghai, China.

### 3.2. Preparation and Isolation of Apple Pectin Oligosaccharides (APOSs)

The preparation and separation methods were mainly referenced from our previous study and the published research of Yeung et al., with some modifications in this work [27,52]. Raw apple pectin (plant-derived fruit pectin, quality grade 100, white to light brown powder) was adopted as the raw material, which possessed a methoxylation degree of 50–75%, impurity ≤ 10% water, and ignition residue ≤ 7.0% (data from Sigma apple pectin 93854). For the preparation reaction, 1.0 g of apple pectin was dissolved in 100 mL deionized water to prepare a 10 mg/mL solution (pH = 2.4–2.5). The pH of the pectin solution was adjusted to 10 using ammonia solution (AR, 25~28%), and the mixture was stabilized at 90 °C for 10 min. Subsequently, a certain amount of hydrogen peroxide (0.68 mL, 30 wt%) was added to the system to obtain a final concentration of 66.18 mM for the oxidation reaction at pressure bottle. The mixture was then shaken (200 rpm) on a water bath shaker at 90 °C for 3 h. Afterwards, the final pH (9.2–9.3) was returned to its initial value using HCl (4 wt%).

To isolate apple pectin oligosaccharides (APOSs), 157.5 mL of anhydrous ethanol was slowly added to the cooled reaction mixture (105 mL) while continuously stirring to achieve a final ethanol concentration of 60% (*v*/*v*). The mixture was sealed and kept at 4 °C for 24 h for static precipitation. Afterwards, the mixture was centrifuged at 8000 r/min for 10 min to remove precipitates. The collected supernatant was concentrated and freeze dried, with a single-pass yield of 38.57%. To ensure sufficient sample dosage and experimental uniformity for subsequent structural characterization and antioxidant tests, multiple batches of products were prepared and homogenized, and the homogeneous powder was reserved for further application and analysis.

### 3.3. Monosaccharide Determination of APOS

Monosaccharide determination of APOS referred to the previous studies [27,53]. APOS (5 mg) and 3 M trifluoroacetic acid (2 mL) were mixed and reacted for 3 h at 120 °C. After nitrogen drying, the dry sample was reconstituted in 5 mL deionized water. The above solution (50 μL) was diluted to 1 mL, centrifuged (12,000 rpm, 5 min), and the supernatant was analyzed via ion chromatography. The sixteen monosaccharide standard solution was prepared using the same method. Chromatographic column: Dionex Carbopac™ PA20 column (3 × 150 mm) at 30 °C, mobile phase: (A) H_2_O, (B) 15 mM NaOH, and (C) 15 mM NaOH/100 mM NaOAc, flow rate: 0.3 mL/min, injection volume: 5 µL, gradient elution (V/V): 0–20 min, 98.8% A/1.2% B; 20.1–30 min, 50% A/50% B; 30.1–46 min, 100% C; 46.1–50 min, 100% B; 50.1–80 min, 98.8% A/1.2% B for column re-equilibration. Detection was carried out using an electrochemical detector. The molar mass of the various monosaccharides was used to calculate the molar ratio, and the amount of each monosaccharide was determined by the absolute quantification method.

### 3.4. SEM Determination of Apple Pectin and APOS

The surface morphology (500× magnification) of apple pectin and APOS was determined using a SU8010 scanning electron microscope (Carl Zeiss AG, Oberkochen, Germany).

### 3.5. FTIR Determination of Apple Pectin and APOS

FTIR analysis (400–4000 cm^−1^) of apple pectin and APOS was conducted with a Tensor 37 Fourier transform infrared spectrometer (Bruker, Ettlingen, Germany) using the potassium bromide (KBr) pellet method.

### 3.6. NMR Determination of APOS

APOSs were characterized via ^1^H NMR and ^13^C NMR spectroscopy (Bruker Avance III HD 400 MHz, Bruker BioSpin AG, Faellanden, Switzerland) using the method referred to in our previous report [27].

### 3.7. UPLC/Q-TOF-MS Determination of APOS

UPLC/Q-TOF-MS detection was performed using a Waters Acquity UPLC instrument (Waters, Milford, MA, USA) and referring to our previous study [27]. Chromatographic column: BEH Amide analytical column (1.7 μm, 2.1 × 150 mm) coupled with a matching Vanguard pre-column (1.7 μm, 2.1 × 5 mm). Column temperature: 35 °C, flow rate: 0.3 mL/min, injection volume: 1 μL, needle washing: strong eluent (acetonitrile–water, 20:80, *v*/*v*) and weak eluent (acetonitrile–water, 75:25, *v*/*v*), mobile phase: solvent A (acetonitrile containing 0.1% formic acid) and solvent B (aqueous solution with 10 mM ammonium acetate and 0.1% formic acid), elution gradient: 0–1 min, 80% A; 1–25 min, 80% to 50% A; 25–30 min, 50% to 80% A; 30–35 min, isocratic elution at 80% A, mode: positive ion, capillary voltage: 3.0 kV, cone voltage: 40 V, ion source temperature: 100 °C, desolvation temperature: 400 °C, cone gas flow 50 L/h, and desolvation gas flow 800 L/h.

### 3.8. Determination of Antioxidant Activity

DPPH Radical Scavenging Assay: DPPH solution was obtained from previous study, with some modified [27,54]. APOS (10.00, 8.00, 3.00, and 1.00 mg/mL, 300 µL) and DPPH solution (300 µL, DPPH absolute ethanol solution, 0.04 g/L) were mixed and reacted at 25 °C for 30 min in the dark, and then measured at 517 nm. The scavenging rate was calculated as:

(1) $$\mathrm{Scavenging}\;\mathrm{rate}\%=(1-\frac{A_{1}-A_{2}}{A_{0}})\;\times \;100\%,$$

where *A*_0_, *A*_1_, and *A*_2_ are the absorbances of the blank, sample test, and control, respectively.

ABTS Radical Scavenging Assay: ABTS diluting solution was obtained from previous study, with some modified [21,27]. APOS (9.00, 7.00, 5.00, and 3.00 mg·mL^−1^, 100 µL) and 100 µL of ABTS diluting solution were mixed and reacted in the dark for 35 min, and measured at 734 nm. The scavenging rate was calculated as:

(2) $$\mathrm{Scavenging}\;\mathrm{rate}\;\%=(1-\frac{A_{1}-A_{2}}{A_{0}})\;\times \;100,$$

where *A*_0_, *A*_1_, and *A*_2_ are the absorbances of the blank, sample test, and control, respectively.

### 3.9. Data Analysis

All experiments were performed in triplicate, and the data were expressed as mean ± standard deviation (SD). One-way ANOVA and Duncan’s test was used to determine significant differences (*p* < 0.05) by IBM SPSS 20.0 software.

## 4. Conclusions

In this study, apple pectin oligosaccharides (APOSs) were successfully prepared using a green and efficient hydrogen peroxide degradation method combined with ethanol precipitation. Structural characterization revealed that APOSs are primarily composed of galacturonic acid (58.2%), galactose (28.6%), arabinose (4.5%), and rhamnose (4.1%), with a low Rha/GalA molar ratio (0.07), indicating that they are mainly derived from the homogalacturonan (HG) region of apple pectin, along with contributions from rhamnogalacturonan-I (RG-I) domains. FTIR and NMR analyses confirmed that the degradation process induced de-esterification, resulting in an increased proportion of free carboxyl groups, while the pyranose skeleton and α-glycosidic configuration were largely retained. UPLC/Q-TOF-MS analysis further identified oligosaccharides with degrees of polymerization up to 8, including methyl-esterified and acetylated galacturonic acid residues. Antioxidant activity assays demonstrated that APOSs possess potent dose-dependent radical scavenging activity, with DPPH scavenging rates reaching 84.6% at 10 mg/mL and ABTS scavenging rates reaching 96.2% at 9 mg/mL. These findings suggest that the hydrogen peroxide degradation method is an effective green approach for producing bioactive APOSs, and that the obtained APOSs hold promise as natural antioxidants for applications in functional foods, pharmaceuticals, and biomaterials. Future work should focus on further fractionation of APOSs based on molecular weight and degree of polymerization to elucidate the precise structure–activity relationships and enhance their targeted antioxidant efficacy.

## Figures and Tables

**Table 1:** Monomeric composition of APOS.

Name	Peak Area	RT	Molar Ration	μg/mg
Rhamnose (Rha)	3.019	12.142	0.041	17.34
Arabinose (Ara)	5.596	13.267	0.045	17.47
Galactose (Gal)	21.521	17.117	0.286	132.11
Glucose (Glc)	2.299	19.525	0.016	7.58
Xylose (Xyl)	2.243	22.984	0.026	10.20
Galacturonic acid (GalA)	21.342	43.184	0.582	289.82
Glucuronic acid (GlcA)	0.252	45.65	0.003	1.48

**Table 2:** The proposed structure of APOS.

*m*/*z*	Ionic Form	Measured Mass (Mass Error)	Proposed Structure	Source
242.29	[M+NH_4_−H_2_O]^+^	242.29 (−0.11)	GalA(Ac)_2_	HG
403.14	[M+H+H_2_O]^+^	384.14 (−0.04)	GalA_2_(Me)	HG
337.23	[M+Na+H_2_O]^+^	296.23 (−0.23)	RhaAra	RG-I
413.29	[M+H+H_2_O]^+^	394.29 (−0.01)	GalA_2_(Ac)	HG
427.25	[M+H−H_2_O]^+^	444.25(−0.25)	GalAra_2_	RG-I
546.48	[M+NH_4_−H_2_O]^+^	546.48 (−0.36)	GalA_3_	HG
562.43	[M+NH_4_]^+^	544.43 (−0.26)	GalA_2_Rha(2Me)	RG-I
637.35	[M+H+H_2_O]^+^	618.35 (−0.14)	GalARha_2_Ara	RG-I
663.45	[M+H]^+^	662.45 (−0.25)	GalA_2_Rha_2_	RG-I
750.54	[M+NH_4_−H_2_O]^+^	750.54 (−0.36)	GalA_4_(2Me)	HG
942.63	[M+NH_4_−H_2_O]^+^	942.63 (−0.35)	GalA_2_RhaGalAra_2_	RG-I
1028.67	[M+NH_4_−H_2_O]^+^	1028.67 (−0.40)	GalA_4_(Me)Rha	HG
1071.69	[M+H−H_2_O]^+^	1088.69 (−0.35)	GalA_2_Rha_2_ GalAra_2_	RG-I
1107.71	[M+H+H_2_O]^+^	1088.71 (−0.42)	GalA_3_RhaAra_3_	RG-I
1194.21	[M+NH_4_]^+^	1176.21 (+0.14)	GalA_3_Rha_2_Gal_2_(Me)	RG-I
1280.24	[M+NH_4_−2H_2_O]^+^	1298.24 (+0.20)	RhaGal_7_	RG-I

Ac: acetylation, Me: methylation.

## Data Availability

The data presented in this study are available on request from the corresponding author.

## Abbreviations

The following abbreviations are used in this manuscript:

POSs	Pectic oligosaccharides
APOSs	Apple pectin oligosaccharides
DP	Degree of polymerization
HG	Homogalacturonan
Rha	Rhamnose
Ara	Arabinose
Gal	Galactose
Glc	Glucose
Xyl	Xylose
GalA	Galacturonic acid
GlcA	Glucuronic acid
